# Combining High-Pressure Perturbation with NMR Spectroscopy for a Structural and Dynamical Characterization of Protein Folding Pathways

**DOI:** 10.3390/molecules25235551

**Published:** 2020-11-26

**Authors:** Cécile Dubois, Isaline Herrada, Philippe Barthe, Christian Roumestand

**Affiliations:** Centre de Biochimie Structurale, INSERM U1054, CNRS UMR 5048, Université de Montpellier, 34090 Montpellier, France; cecile.dubois@cbs.cnrs.fr (C.D.); isaline.herrada@yahoo.fr (I.H.); Philippe.Barthe@cbs.cnrs.fr (P.B.)

**Keywords:** protein folding, NMR, High Hydrostatic Pressure

## Abstract

High-hydrostatic pressure is an alternative perturbation method that can be used to destabilize globular proteins. Generally perfectly reversible, pressure exerts local effects on regions or domains of a protein containing internal voids, contrary to heat or chemical denaturant that destabilize protein structures uniformly. When combined with NMR spectroscopy, high pressure (HP) allows one to monitor at a residue-level resolution the structural transitions occurring upon unfolding and to determine the kinetic properties of the process. The use of HP-NMR has long been hampered by technical difficulties. Owing to the recent development of commercially available high-pressure sample cells, HP-NMR experiments can now be routinely performed. This review summarizes recent advances of HP-NMR techniques for the characterization at a quasi-atomic resolution of the protein folding energy landscape.

## 1. Introduction

Since Anfinsen’s early works [1], much effort has been expended in attempting to understand how amino acid sequence impacts the structure, dynamic properties, and global stability of proteins. On the other hand, much less is known about how proteins fold from single, disordered inactive polypeptide chains to unique tri-dimensional active structures. General descriptions of folding pathways cannot be predicted for arbitrary amino acid sequences but can be reached only from experimental studies, possibly coupled to molecular dynamic simulations. Thus, revealing the folding mechanism of proteins required knowledge from different fields: biology but also chemistry and physics, including computational simulations. Finally, the theoretical framework of free energy landscape theory and the funnel concept emerged [2,3,4,5,6,7,8], giving satisfactory models to understand this mechanism.

Folding/unfolding experiments performed in vitro have yielded much of the information concerning protein folding mechanisms. To this aim, several perturbation methods have been used, among them the addition of chemical denaturants (urea, guanidinium chloride), pH changes, or modification of the temperature of the sample are the most popular. Alternatively, pressure presents a lot of advantage to study protein unfolding. First, unfolding by pressure is reversible, essentially because high pressure, contrary to temperature, disfavors intermolecular protein interactions, preventing irreversible aggregation. This reversibility allows the measurement of thermodynamic parameters for the folding/unfolding reaction [9,10,11]. Second, contrary to the use of pH or chaotropic reagents, it does not change the charge or the chemical composition of the system. Pressure is actually a very straightforward perturbation: it induces unfolding because the molar volume of the folded states is larger than that of the unfolded states of protein, essentially because of the existence of solvent excluded voids in the folded states that are eliminated in the unfolded states [12,13]. Thus, following the Le Chatelier’s principle, pressure shifts the folded state/unfolded state equilibrium toward the unfolded state, the one with the lower molar volume.

Pressure perturbation is generally used in combination with circular dichroism [14], fluorescence [15], or FT-IR spectroscopy [16,17,18]: these spectroscopic methods give global information on the state of the system, i.e., on the relative populations of the folded and unfolded states at a given pressure. Due to their extreme sensitivity to the structural environment, Nuclear Magnetic Resonance (NMR) observables constitute an attractive alternative. Global information on protein folding reaction can be obtained from simple 1D NMR spectra, as with the other spectroscopies quoted before. But more interestingly, multi-dimensional (2D) NMR provides multiple probes in the protein structure, giving access to local, residue-specific information. Thus, at the expense of some difficulties in implementation, most of which have been adequately addressed in commercial instrumentation, the combination of high-pressure perturbation with multi-dimensional NMR constitutes a powerful tool that can be used to describe the conformational landscape of proteins at a resolution which cannot be accessed by global spectroscopic observables.

## 2. High Pressure NMR Instrumentation

Combining high pressure with NMR spectroscopy constitutes a real challenge since several difficulties must be overcome: the system should be pressure-resistant, permeable to radiofrequency used for spin excitation, and non-magnetic in order to be safely introduced in the magnet. Of course, the conventional borosilicate or Pyrex glass tubes currently used for high-resolution liquid-state NMR do not match the “pressure-resistant” criterion, even though some commercial manufacturers offer borosilicate glass tubes, which can support moderate pressure up to 12 bar (Norell^TM^). These tubes are essentially designed to work with gas-pressurized samples, the pressure limit being too low to allow protein denaturation, usually expected in the range 1–12 kbar at ambient temperature [19].

The first set-up really adapted for high-pressure NMR spectroscopy was the “autoclave” system developed by Benedek & Purcell in 1954 [20]. In this set-up, the sample and the radiofrequency coil are directly placed in a high-pressure non-magnetic vessel made initially of beryllium–copper alloy, later replaced by more resistant titanium alloy [21,22,23,24]. Very high pressure can be obtained with this set-up (9–10 kbar), compatible with protein denaturation. Nevertheless, it suffers from different drawbacks: among other things, the electrical coupling between the coil and the metallic chamber alters the coil efficiency, yielding low sensitivity. In addition, adding a second coil in the chamber is difficult, impeding the realization of heteronuclear experiments, standard in biomolecular NMR. An original alternative was proposed by Castro and Delsuc [25], where the metallic chamber was replaced by a composite material chamber of fiberglass and epoxy resin. Due to the insulator property of this material, the proton radiofrequency coil (excitation and detection) can be directly embedded in the chamber wall, and an additional coil for heteronuclei excitation can be glued directly on the external surface of the chamber. Nevertheless, the use of this probe was limited by its low burst pressure (1.5–2 kbar), hardly compatible with protein denaturation. Very recently, Meier et al. [26] published what can be considered as the ultimate development of the autoclave approach, at least in terms of pressure limit. They replaced the titanium chamber with a diamond anvil cell (DAC), similar to those used for HP crystallography [27]. The sample and the resonator are placed directly in the DAC, and pressures up to 0.9 Mbar can be reached with this set-up. Nevertheless, this set-up is not adapted to biomolecular RMN: the design of the radio-frequency section is not adapted to biomolecular NMR, and the sample volume (about 100 pL) is too small to yield enough sensitivity in case of biomolecules. Moreover, such very high pressure is not really useful for the study of protein denaturation. On the contrary, at above 10 kbar changes in the water structure are expected, which hamper the thermodynamic analysis.

The alternate strategy to “autoclave” systems is pressure-resistant tubes or cells that limit the pressurized region to the sample itself and can be used with commercial NMR probes. Polyimide (Vespel) [28] and single crystal sapphire tubes [29] that can withstand pressure up to 1 kbar were proposed. If they improved comfort in the use of HP-NMR by organic chemists, their low burst pressure makes them unsuitable for the study of protein denaturation. In the mid-1970s, Yamada et al. [30,31] developed high-pressure glass (or quartz) cells allowing to work with pressure up to 3 kbar. Even if this pressure seems too low to enable denaturation for many proteins, this drawback can be circumvented by adding sub-denaturing concentration of chaotropic reagents [32] or of organic solvents to the buffer, and playing with the temperature [33], in order to tune the protein stability with the pressure range allowed by the system. The set-up consists of a long capillary enlarged on one end to form the cell itself that goes into the NMR probe, protected by a Teflon tube. The other end of the capillary is glued on a bronze–beryllium valve that allows pressure transmission from the HP-pump. The capillary length should fit with the magnet size, in order to be long enough to maintain the bronze–beryllium seal far from the magnetic center of the magnet, minimizing perturbations of the magnetic field homogeneity, thus permitting to record high-resolution spectra. It can be used with any commercial NMR probes and allows recording any through-bond or through-space homonuclear or heteronuclear (double-, triple-resonance) correlation NMR experiments. Akasaka and coworkers have used this cell to characterize the folding of numerous globular proteins [10]. Nevertheless, the manufacturing of this system remains delicate, and the sensitivity of the NMR experiments is limited by the small sample volume available (30–40 µL), requiring highly concentrated samples [34,35]. In 1996, Wand and coworkers developed a new set-up consisting of a simple two-component valve system that holds and seals a high-pressure sapphire tube [36,37]. A similar and complementary approach was proposed by the group of Kalbitzer [38], initially based on a sapphire tube and later replaced by a ceramic tube [39]. The later developments of these set-ups [40] have been integrated in the system now commercially available from Deadalus Innovation^TM^ company. The high-pressure sample tubes are made from aluminum-toughened zirconia ceramic. They provide access to pressures up to 3 kbar and to a −15 to 115 °C temperature range. With an outer diameter of 5 mm, they are compatible with most of the commercially available probes. The inner diameter of 2.8 mm provides a working volume of about 200 µL, which allows for a sensitivity near that of 3 mm glass tubes, standard now for (ambient pressure) biomolecular NMR at high fields. The two-component valve initially proposed by Urbauer and Wand [36,37] is used to couple the ceramic tube to the high-pressure tube. Compared with Yamada’s cell, this set-up provides a similar spectral quality but about a 10-fold increase in sensitivity and is incomparably easier to handle, essentially due to a wider inner diameter facilitating its filling. Thus, these ceramic tubes can be easily filled with complex viscous samples, such as those used for RDC measurement [41,42,43]. Pressure is generally transmitted by mineral oil, so that no physical separation is needed between the aqueous buffer containing the protein and the transmitting fluid.

## 3. “Global” Thermodynamic and Kinetic Parameters for Folding/Unfolding Reactions Obtained from 1D High-Pressure NMR Spectroscopy

Typically, 1D HP-NMR can be used for steady-state measurements, recorded when the ratio between folded/unfolded protein populations has reached equilibrium after a pressure jump. Such measurements give access to the global thermodynamic parameters ∆*G*^0^ (the free-energy difference at atmospheric pressure between the folded and unfolded states of the protein) and ∆*V*^0^ (the volume difference at atmospheric pressure between the folded and unfolded states of the protein), characteristic of the folding/unfolding reaction. In addition, kinetic measurements recorded just after the *P*-jump, while establishing equilibrium, are also possible with HP-NMR, yielding kinetic information on the folding/unfolding reaction as well as volumetric properties of protein folding transition states.

### 3.1. Steady-State Measurements of Global Thermodynamic Parameters with 1D High-Pressure NMR 

**Figure 1:**
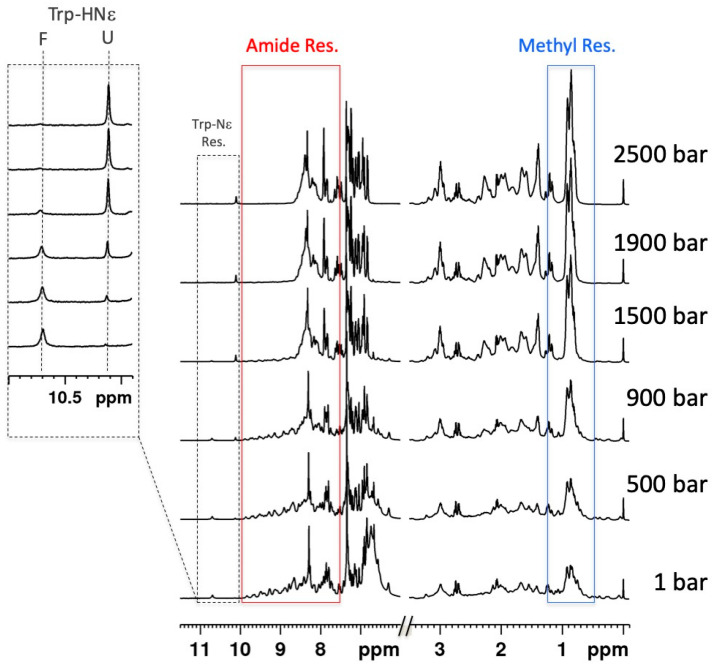
Evolution upon pressure of the 1D ^1^H-NMR spectrum of Titin I27 Ig-like domain. Stacked plot of 1D spectra recorded as a function of pressure at 600 MHz and 298 K on a 1 mM sample of Titin I27 in Tris buffer pH 7.0, 1 mM DTT. A 1.7 M sub-denaturing concentration of GuHCl has been added to the sample in order to decrease the protein stability and to observe complete unfolding in the 1–2500 bar pressure range allowed by the experimental set-up (zirconium oxide ceramic tubes, Daedalus Innovation^TM^). The solid-line frames delimit the regions corresponding to HN amide (red frame) and CH_3_ methyl group resonances (blue frame). The insert corresponds to a zoom on the indole resonances region (black dashed-line frame) showing the decrease with pressure of the HN indole resonance of Trp-34 in the folded state (F) and the concomitant increase of the same resonance in the unfolded states (U).

The good dispersion usually observed in the ^1^H-NMR spectrum of a folded protein is essentially due to the extreme sensibility of the proton NMR resonances (or chemical shifts) to through-space effects of neighboring groups. These effects vanished when the protein unfolds, and the ^1^H-NMR spectrum becomes poorly resolved. For instance, the well-resolved regions characteristic of resonances of the methyl groups, or of the amide groups, in the ^1^H spectrum of a folded protein will collapse upon unfolding (Figure 1). 

Thus, the evolution of the 1D NMR spectrum allows us to monitor the high-pressure denaturation of a protein, as depicted in Figure 1 for the I27 Immunoglobin-like domain of the sarcomeric protein Titin [44]. As an effect of the energy barrier between the folded and unfolded protein (≈2 kcal/mol in the experimental conditions reported in Figure 1), these species are in slow exchange with regard to the NMR timescale: we observe the disappearance of resonances belonging to the native state, with the concomitant appearance of new peaks that correspond to the spectrum of the unfolded states. 

**Figure 2:**
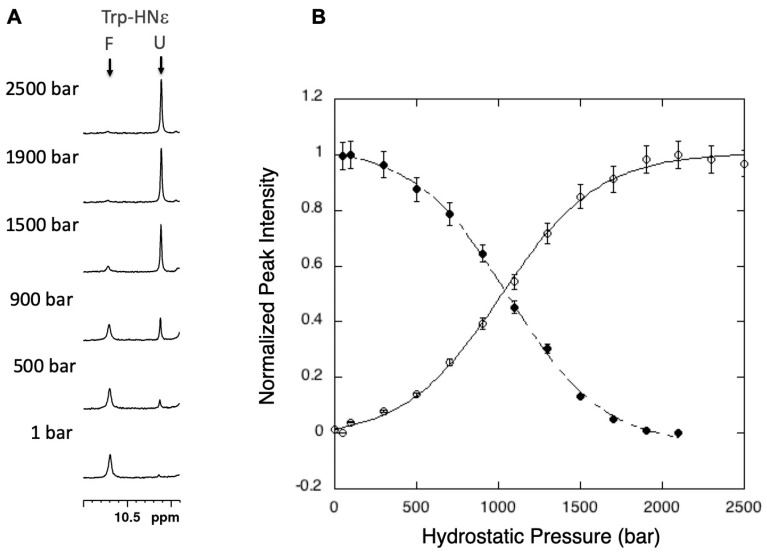
Monitoring the unfolding reaction of titin I27 domain with 1D HP-NMR spectroscopy (**A**): 1D HN indole region of the proton NMR spectra of Titin I27 recorded at increasing pressure. F stands for the resonance in the folded state, U for the resonance in the unfolded state. (**B**): denaturation curves obtained from the fit of the evolution with pressure of the native (open circle) and the denatured (filled circle) indole resonance of tryptophan-34 with a two-state equilibrium equation. Similar values of ∆*V*^0^ are found for both fits.

As it can be observed for the indole resonance of Trp-34 (Figure 2), the decrease in pressure of the peak corresponding to the folded (F) state, as well as the increase of the peak corresponding to the unfolded states (U), can be generally well-fitted by a sigmoidal curve [45,46] characteristic of a two-state equilibrium in the form of:

with a characteristic $F\;\overset{K_{u}}{\underset{K_{f}}{⇌}}\;U$ equilibrium constant of:

*K*_eq_ = *k_f_*_(p)/_*k_u_*_(p)_ = [U]/[F] (1)

where *k*_f(p)_ and *k*_u(p)_ stands for the folding and the unfolding rate constants at a given pressure p. *K*_eq_ can be also expressed from the Boltzmann equation as:

*K*_eq_ = exp(−∆*G*_eq_/R*T*) (2)

where the free energy change can be expressed as a Taylor expansion, truncated at the second order term:

∆*G*_eq_ = *G*_U_ – *G*_F_ = ∆*G*^0^ + ∆*V*^0^(p − p_0_) – ½ ∆β*V*^0^(p − p_0_)^2^ (3)

Here ∆*G*_eq_ and ∆*G*^0^ are the Gibbs-free energy changes from F to U at pressure p and p_0_ (p_0_ = 1 bar), respectively; ∆*V*^0^ is the partial molar volume change; ∆β is the change in compressibility coefficient (∆β = −(1/*V*^0^) * δ*V*/δp), R is the gas constant, and *T* is the absolute temperature. It has been shown that for proteins the difference in compressibility between native and denatured states is negligible [47]. Thus, the expression of ∆*G*_eq_ simplifies to:

∆*G*_eq_ = ∆*G*^0^ + ∆*V*^0^(p − p_0_) (4)

Using NMR spectroscopy, the observable will be *I*, either the intensity (peak height) or the integral of a peak corresponding to either the folded species or of the unfolded species. In the present case (Figure 2), we chose to follow either the decrease of the peak intensity corresponding to the HN indole resonance of Trp-34 in the folded species or the increase of the corresponding resonance in the unfolded species. Alternatively, one can follow the increase of the peak at 0.86 ppm corresponding to the resonances of methyl groups in the unfolded species. Thus, the equilibrium constant can be written as:

(5) $$K_{eq}\;=\;\frac{\left[U\right]}{\left[F\right]}\;=\;\frac{I_{F}-I}{I-I_{U}}$$

If we choose to follow the increase with pressure of a resonance corresponding to the unfolded species in the 1D NMR spectrum, *I_F_* stands for the intensity of the corresponding NMR line in the folded spectrum at 1 bar (*I_F_* = *I_min_*), whereas *I_U_* corresponds to the intensity of the same line at high pressure, when the protein is fully unfolded (*I_U_* = *I_max_*). Combining this equation with Equations (2) and (4) gives the characteristic equation for a two-state equilibrium:

(6) $$I\;=\;\frac{I_{F}+I_{U}e^{-\left[\Delta G^{0}\;+\;\left(p\;-\;p0\right)\Delta V^{0}\right]/RT}}{1+e^{-\left[\Delta G^{0}\;+\;\left(p\;-\;p0\right)\Delta V^{0}\right]/RT}}$$

Fitting either the sigmoidal decrease with pressure of the indole resonance in the folded state or the sigmoidal increase of the indole resonance in the unfolded state (Figure 2) yield “global” values for ∆*V*^0^ of unfolding (−84 ± 5 mL/mol and −82.4 ± 5 mL/mol, respectively) and for ∆*G*^0^ (2.14 ± 0.12 kcal/mol and 2.01 ± 0.13 kcal/mol, respectively), under the conditions of the study (pH = 7, 25 °C, 1.7M GuHCl). Note that the values extracted from the two different fits fall within experimental uncertainties, confirming the two-state equilibrium for the folding/unfolding reaction. Slightly different values (∆*V*^0^ = −78.8 ± 4 mL/mol, ∆*G*^0^ = 2.26 ± 0.26 kcal/mol) can be measured at equilibrium when referring to the resonance corresponding to methyl groups in the unfolded states (0.96 ppm). This indicates that we are measuring “apparent” values for these thermodynamic parameters that depend of course on the global stability of the protein but that are also influenced by the local stability sensed by a given resonance in a given environment. As we will see further, this is of paramount importance for the description of protein folding pathways.

### 3.2. Measurements of Global Kinetic Parameters of the Folding/Unfolding Reaction with 1D High-Pressure NMR 

∆*V*^0^ and ∆*G*^0^ are thermodynamic parameters at atmospheric pressure, characteristics of the system at equilibrium. But a kinetic analysis of the folding/unfolding reaction is needed to obtain information on the transition state (usually described not as a unique conformer but as an ensemble of conformers, hence the term of “Transition State Ensemble” (TSE) used for proteins) of the reaction. Even though characterizing transition states in protein folding constitutes an essential step in the puzzle [48], the relations between the protein sequences, their 3D structures, and the structure (at least the hydration state) of their TSE are not yet well understood. Thus, the HP-NMR comparative study of the folding of Titin I27 module and DEN4-ED3 domain from the viral envelope of the dengue virus, two proteins with unrelated sequences but sharing a common Ig-like fold, shows similar folding intermediates but very different TSE, the transition state of Titin I27 being considerably less hydrated than the one of DEN4-ED3 [49]. Such analysis relies on the measurement of kinetic parameters after perturbation (*P*-jump) of the thermodynamic equilibrium between the folded and unfolded conformers of the protein at a given pressure, yielding the rates of folding and unfolding at atmospheric pressure. Moreover, these studies give access to the values of the activation volume between the folded or unfolded state and the TSE, related to the hydration state of the TSE. 

**Figure 3:**
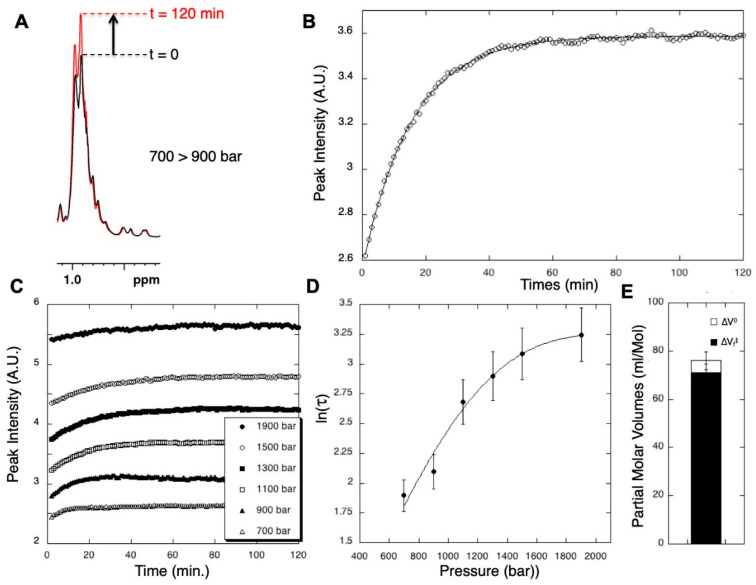
Measuring global kinetic parameters for the folding/unfolding reaction with real-time 1D HP-NMR spectroscopy. (**A**) Two 1D NMR proton spectra (methyl groups resonances) recorded on Titin I27 (same conditions as in Figure 1) just after a 700 to 900 bar P-Jump (black trace) and 2 h after the P-jump (red trace) 1). These two spectra represent the extreme points of a series of sixty spectra of 2 min each recorded over a period of 2 h. The arrow indicates the increase of the resonance at 0.96 ppm that corresponds to methyl groups in the unfolded species. (**B**) Measurement of the relaxation time, τ, at 900 bar, through the fit of the exponential growth of this methyl resonance. (**C**) Exponential growths of the resonance corresponding to the methyl groups in the unfolded states after successive 200 bar P-Jumps between 300 and 1900 bar, the pressure range where Titin I27 unfolds. Relaxation times τ_(p)_ can be measured from these experiments for the different pressures. (**D**) “Chevron plot” of ln(τ) measured at different pressures: the fit with Equation (8) allows to extract the folding or unfolding kinetic rate constants k_f0_ and k_u0_, respectively, and the activation volume of folding $\Delta V_{f0}^{‡}$ or of unfolding $\Delta V_{u0}^{‡}$ at atmospheric pressure. (**E**) Volumetric diagram obtained for Titin I27 domain, displaying average values of $\Delta V_{f0}^{‡}$ (plain bar) and ∆*V*^0^ (open bar). The value of the ratio $\Delta V_{f0}^{‡}$ /∆*V*^0^ close to 1 deduced from this diagram indicates a dehydrated TSE.

Due to the very large volumes of activation involved in the folding/unfolding reaction, high pressure can considerably slow down the rate of folding and also possibly unfolding [13,50]. Thus, although the completion of a folding/unfolding reaction is usually a few seconds at atmospheric pressure, it can take up to a few hours at high pressure (about 12 h for ∆ + PHS SNase at pressure above 1 kbar [12]). This is more than enough for the use of real-time 1D NMR to follow the folding/unfolding reaction after a *P*-jump, until steady state is achieved. Real-time 1D NMR spectroscopy consists in recording with time a series of 1D NMR spectrum at a constant repeating rate. After a *P*-jump, it is then possible to observe the exponential decrease with time of a resonance corresponding to the folded species, or the exponential growth with time of a resonance corresponding to the unfolded states, until the steady state is reached. In the case of Titin I27, we observed the exponential growth with time of the resonance at 0.96 ppm corresponding to the methyl groups of the unfolded states (Figure 3A). Alternatively, the exponential decrease with time after a positive *P*-jump of the well-resolved resonances of the shielded methyl protons can be used as a probe for such measurements, giving residue-specific information on the folding kinetics related to the local hydration of the TSE (see further) [32].

Concerning the experimental aspects for the realization of the *P*-jump, these experiments are more demanding than those used for the steady-state analysis. Indeed, the time needed for the sample pressurization should be negligible with respect to the time needed by the folding/unfolding reaction to reach the plateau. For instance, the 200 bar *P*-jumps used for acquiring the data presented in Figure 3 needed about 10 s when performed with the Deadalus Innovation^TM^ Xtreme electric HP-pump. In this particular case, the time needed to reach the steady state after equilibrium was about 40 min (Figure 3C), so that the pressurization time can be safely neglected. For proteins with shorter folding relaxation times, Kremer et al. [51] have circumvented this limitation by pre-pressurizing a reservoir, upstream of the high-pressure cell, containing a large volume of mineral oil, much greater than the volume corresponding to the pressurization line and the high-pressure cell itself. Thus, opening an electric valve placed in between the reservoir and the cell allows an almost immediate (in the millisecond range) equilibration of the pressure between the reservoir and the sample cell. Since the volume of pressurization liquid is far greater in the reservoir than in the rest of the set-up, the final pressure reached in the sample cell when opening the valve is virtually the initial pressure in the reservoir.

The relaxation time characteristic of the kinetics after a given *P*-jump (τ _(p)_ = 1/(*k*_u(p)_ + *k*_f(p)_), where *k*_u(p)_ and *k*_f(p)_ are the unfolding and the folding rates at the pressure p reached at the end of the *P*-Jump, can be extracted from the fit of the exponential growth of this resonance (Figure 3B). Then, it becomes possible to extract the values at atmospheric pressure of *k*_u0_ and *k*_f0_, as well as those of the activation volume of unfolding $\Delta V_{u0}^{‡}$ (or folding, $\Delta V_{f0}^{‡}$), by measuring this relaxation time after different p-jumps, between different pressures in the range where the protein unfolds (Figure 3C):

At a given pressure: *τ*_(p)_ = 1/(*k*_u(p)_ + *k*_f(p)_)

with $k_{f}\left(p\right)=k_{f0}e^{-p\Delta V_{f0}^{‡}/RT}$ and $k_{u}\left(p\right)=k_{u0}e^{-p\Delta V_{u0}^{‡}/RT}$

*τ*_(p)_ can be rewritten as:

(7) $$\tau_{\left(p\right)}={\left[k_{u0}e^{\left(\frac{-p\Delta V_{u0}^{‡}}{RT}\right)}+\;k_{f0}e^{\left(\frac{-p\Delta V_{f0}^{‡}}{RT}\right)}\right]}^{-1}$$

The value of ∆*V*^0^, the volume difference between the folded and unfolded states measured at equilibrium ∆*V*^0^ (= ∆*V*_f_ − ∆*V*_u_), and *K*_eq_ (= *k*_f0_/*k*_u0_) can be measured from the steady state experiments described above. One can then decrease the number of parameters for the fit:

(8) $$\tau_{\left(p\right)}={\left[k_{u0}e^{\left(\frac{-p\Delta V_{u0}^{‡}}{RT}\right)}+\;k_{u0}K_{eq}e^{\left(\frac{-p\left(\Delta V^{0}\;+\;\Delta V_{u0}^{‡}\right)}{RT}\right)}\right]}^{-1}$$

Only two variables need to be fitted: *k*_u0_ (or *k*_f0_), the unfolding (or folding) rate at atmospheric pressure, and $\Delta V_{u0}^{‡}$ (or $\Delta V_{f0}^{‡}$), the activation volume for unfolding (or folding) at atmospheric pressure. 

The fit is usually performed on a plot of ln(τ) as a function of pressure, displaying the characteristic “chevron plot” pattern (Figure 3D). In the case of Titin I27, the activation volume $\Delta V_{f0}^{‡}$ is close to the equilibrium ∆V^0^ value (Figure 3E), suggesting a dehydrated TSE where most of the native voids are present. 

## 4. “Local” Thermodynamic and Kinetic Parameters for Folding/Unfolding Reactions Obtained from 2D High-Pressure NMR Spectroscopy

In most of the studies reported in the literature, the folding/unfolding reaction of a protein is approximated by a two-state model, excluding the existence of folding intermediates in its folding energy landscape. This is in fact a rough approximation: following the more appropriate model of foldons [52,53], most globular proteins should deviate from a two-state folding mechanism by populating folding intermediates. This apparent discrepancy comes from the very low population of the intermediate states at equilibrium, due to their low stability, which hampers their detection by the usual spectroscopic methods. In addition, spectroscopies often focus on only one observable (intrinsic fluorescence of a tryptophan residue, methyl NMR resonance, etc.), yielding values for the thermodynamic parameters that are supposed to reflect the global stability. We have seen previously that these values are also affected by the local stability of the protein: in the case of Titin I27, significantly different values were obtained for ∆*V*^0^ and ∆*G*^0^ from the pressure dependence of the resonances corresponding to either the unfolded state methyl groups or to the indole NH of the tryptophan side chain. Thus, a better description of the protein folding energy landscape, including the identification of folding intermediates, can be obtained by a multiple probes analysis of the folding process.

Multidimensional homo- or heteronuclear NMR spectroscopy provides an intrinsic multi-probe approach yielding residue specific information, through correlation spectroscopy involving nuclei located on the peptide backbone (^1^H and ^15^N of amide groups, ^1^Hα and ^13^Cα). Amide protons offer ideal probes to monitor the unfolding reaction: each amino acid bears an NH group, with the exception of proline, generally a minority residue in the composition of soluble proteins (< 3%), and will give rise to a specific correlation in the usually well-resolved 2D [^1^H-^15^N] HSQC spectrum of the native protein. In addition, proton/deuteron exchange measurement for amide protons has been extensively used to evaluate the local stability of a protein [54], bringing important information on local unfolding phenomena.

### 4.1. Measurement of “Local” Thermodynamic Parameters for Folding/Unfolding Reaction: Tracking Folding Intermediates in the Protein Energy Landscape with 2D High-Pressure NMR 

**Figure 4:**
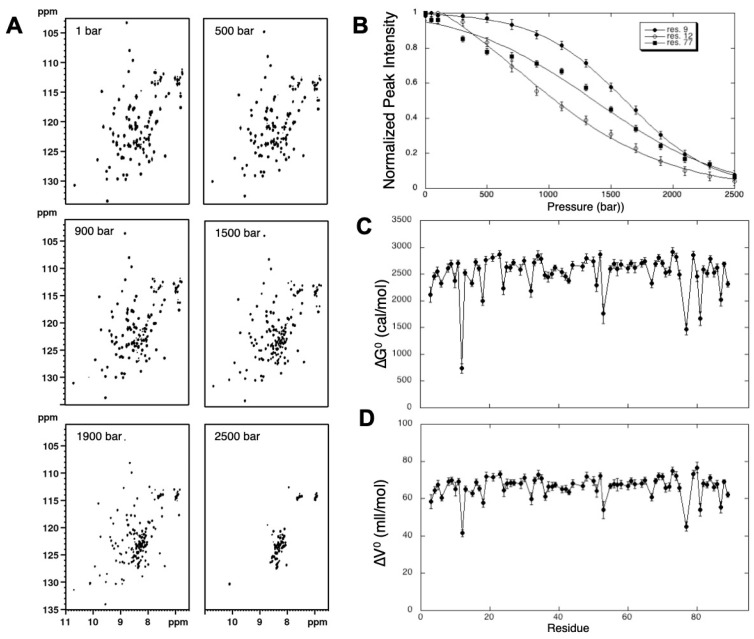
Monitoring unfolding of Titin I27 domain with high pressure 2D NMR. (**A**) Examples of [^1^H-^15^N] HSQC-NMR spectra recorded on a ^15^N-labeled sample of Titin I27 at different pressures as indicated (same other experimental conditions as in Figure 1); (**B**) Overlay of 3 different residue-specific pressure denaturation curves obtained from the fit with Equation (6) of the cross-peak intensities measured at equilibrium from the corresponding residues. For clarity, the cross-peak intensities have been normalized. (**C**) Residue-specific ∆*G*^0^ and (**D**) ∆*V*^0^ measured from residue-specific pressure unfolding curves of Titin I27 domain.

Figure 4 displays the evolution of the correlation peaks on [^1^H,^15^N] HSQC experiments recorded with increasing pressures. According to the slow exchange regime between the folded and unfolded species, already visible and discussed for 1D NMR spectra, one observes the disappearance of correlation peaks belonging to the native form with the concomitant appearance of new peaks (centered at 8.5 ppm on the proton chemical-shift axis), which correspond to the spectrum of the unfolded species. As discussed above, the weak spectral dispersion of the cross-peaks corresponding to the unfolded protein is due to the loss of the through-space effects in the “random coil-like” structure of the unfolded states. 

It is then possible to measure the evolution with pressure of either the intensity (peak height) or the volume of each cross-peak in the HSQC 2D spectrum: for instance, the loss of intensity of the native state resonances directly reflects the decrease in population of the folded state as detected locally by each residue. Note that, although global unfolding of a protein can obey complex models, locally the loss of the native state cross-peak intensity represents a two-state transition, that can be safely fitted with Equation (6). Thus, the fit of the local pressure unfolding curves yields residue-specific values for the apparent volume change (∆*V*^0^) and apparent free energy (∆*G*^0^) difference between the folded and unfolded states (Figure 4C,D).

Note that one usually prefers to fit the sigmoidal decay of the native resonances rather than the sigmoidal growth of the unfolded resonances, even though similar results should be obtained, as mentioned above for the indole resonance of the tryptophan residue (see Figure 2). This is because of the considerably better spectral resolution observed in the HSQC spectrum of the folded protein, which is also usually assigned, contrary to the spectrum of the unfolded states.

Large variations in the Δ*V*^0^ and Δ*G*^0^ values within the protein sequence sign deviation from a simple two-state unfolding transition and suggest the potential presence of folding intermediates. For instance, in the case of Titin I27, whereas a ∆*V*^0^ for unfolding of ≈ −70 mL/mol was measured for most of the residues, ∆*V*^0^ fell to a value < 55 mL/mol for some residues, meaning that some regions of the protein unfold earlier than others and suggesting the presence of partially folded intermediates in the protein energy landscape with some degree of stability (Figure 4). In this particular case, 2D HP-NMR clearly revealed the existence of a folding intermediate where the N-terminal β-strand is detached from the Ig-like β-sandwich. This intermediate was generally not detected in chemical denaturation studies [55] and only suspected in force spectroscopy studies [56,57] of Titin I27 multi-modules constructs. This is a clear demonstration of the potency of HP-NMR that can bring unprecedented details in the analysis of protein folding pathways.

**Figure 5:**
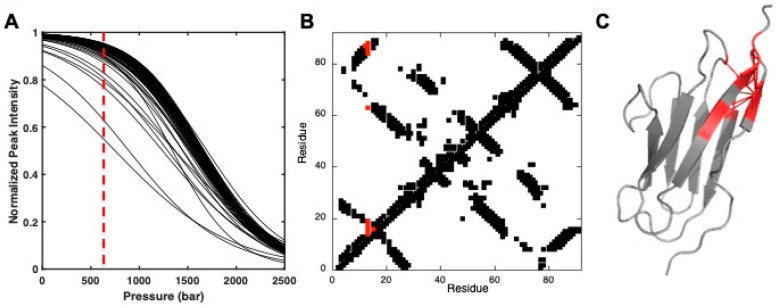
Pressure denaturation of Titin I27 domain. (**A**) Overlay of the normalized residue-specific denaturation curves obtained for Titin I27 domain. The vertical dashed red line at 600 bar represents the pressure used for analysis of the data presented here. (**B**) Contact map built from the best solution structure obtained for Titin I27 Ig-like domain [44]. All native contacts are displayed below the diagonal, whereas only native contacts for which a probability can be calculated from corresponding residue-specific denaturation curves are presented above the diagonal. In addition, the contacts above the diagonal have been colored in red when contact probabilities *p*(ij) lower than 0.5 are observed at 600 bar. (**C**) Ribbon representations of the solution structure of I27 where the red sticks represent contacts that are weakened (*p*(ij) ≤ 0.5) at 600 bar. Residues involved in these contacts are also colored in red on the ribbon.

Structural information on the folding intermediates can also be obtained from residue-specific denaturation curves [12,58,59]. To this aim, the residue specific curves must be first normalized (Figure 5). Then, at a given pressure, the value of 1 measured for a given cross-peak (*I* = *I_F_* = 1) can be associated with a probability of 1 (100%) to find the corresponding residue “i” in the native state, whereas, at the same pressure, a residue “j” for which the corresponding cross-peak has disappeared (*I* = *I_U_* = 0) from the HSQC spectrum has a probability equal to zero to be in a native state.

Considering now a pressure where these two residues i and j are in an intermediate situation where the probability to be in a folded state are *p*(i) and *p*(j) (0 < *p*(i) and *p*(j) < 1), if these two residues are in contact in the native state (at atmospheric pressure) their probability *p*(i,j) to be in contact at this pressure is given by the geometric mean of the two individual probabilities: $p\left(i,j\right)\;=\;\sqrt{p\left(i\right)\times p\left(j\right)}$ [60]. These contact probabilities can be displayed with a color code on contact maps constructed from the 3D crystal or NMR native structure of the protein, by measuring all contacts (usually only those concerning Cα atoms of the different residues, for simplicity) between different atoms (Figure 5). When combined with molecular dynamic simulations, this approach can give a pictorial representation of the conformational ensemble. To this aim, native contact lists generated from contact maps and weighted by the probabilities of contact *p*(ij) at a given pressure are used in Go-model simulations in order to generate multiple conformers and to possibly solve the structure of folding intermediates [12].

Beside this now well-established method, the use of the pressure dependence of amide exchange rates was proposed to characterize intermediate states. Again, a residue-specific measurement of amide exchange rate constants can be obtained from the decrease in intensity of their corresponding cross-peak in the [^1^H-^15^N]-HSQC after dissolving a lyophilized protein sample in D_2_O buffer. The use of H/D exchange measurements [54] has been proposed to identify local stabilities in globular proteins [61,62] through the values of individual amide protection factors (PF) calculated from the experimental exchange rate constants [63]. Note that the values of PF strongly depend on the physical and chemical parameters of the system: pH, temperature, and also pressure [63,64]. 

H/D exchange experiments combined with pressure perturbation have been used for the first time to examine the energetics of apocytochrome b562 [64]. With increasing pressure, a systematic decrease in the protection factors was observed, and changes on apparent volume for exchange (∆*V*_ex_) were estimated from the linear dependence of the free energy of exchange with pressure (Δ*G*_ex_(p) = Δ*G*_ex_^0^ + pΔ*V*_ex_). Three regions with distinct stabilities and pressure sensitivities can be identified [64]. We have used this method for ∆+PHS SNase and several of its cavity mutants and found results in good agreement with our previous equilibrium unfolding data [65]. Nevertheless, one limitation of this method is that it applies only to solvent protected amide protons, under conditions where H/D exchange rates are still measurable (relatively low pH and low temperature).

### 4.2. Measurements of “Local” Kinetic Parameters of the Folding/Unfolding Reaction with 2D High-Pressure NMR

**Figure 6:**
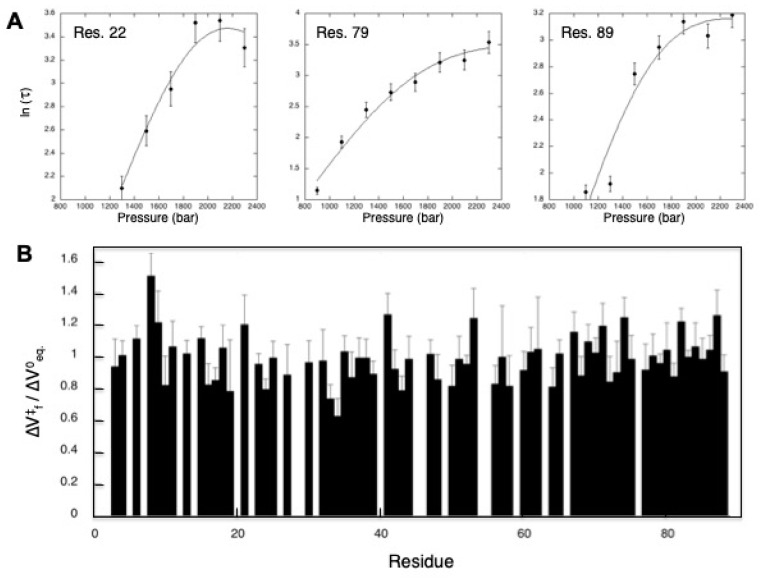
Residue-specific analysis of the unfolding reaction kinetics of Titin I27 domain. (**A**) Examples of residue-specific chevron plots measured for Titin I27 domain. The residue-specific relaxation times τ have been extracted from the decay with time of the intensity of cross-peaks belonging to the native protein species in a series of sixty 2D [^1^H-^15^N]- SOFAST-HMQC experiments (2 min measuring time each) recorded during 2 h after P-jumps of 200 bar. (**B**) Residue-specific values for the ratio $\Delta V_{f0}^{‡}$/∆V^0^ deduced from the fit of residue-specific chevron plots with Equation (6) and plotted versus the sequence of Titin I27.

As for the steady-state parameters ∆*V*^0^ and ∆*G*^0^ discussed above, 2D real-time high-pressure NMR can allow a residue specific analysis for the kinetic parameters of the folding/unfolding reaction: the values of the unfolding and folding rate constants *k*_u0_ and *k*_f0_, as well as the value of the activation volume of unfolding $\Delta V_{u0}^{‡}$ (or folding, $\Delta V_{f0}^{‡}$). This can be readily done by following the exponential decrease in intensity (or volume) after a *P*-Jump of cross-peaks corresponding to the native protein in a series of 2D [^1^H,^15^N] HSQC spectra recorded with time. These experiments can allow a residue-specific description of the TSE, with the location of internal voids already formed at this step of the folding reaction. In other words, they provide a structural description of the TSE, with the location of the “dry” folded regions ($\Delta V_{f0}^{‡}$/∆V^0^ close to 1) and of the hydrated unfolded ones ($\Delta V_{f0}^{‡}$/∆V^0^ close to 0) (Figure 6).

Nevertheless, the time resolution of NMR spectroscopy is limited: the recording time of a 2D [^1^H,^15^N] HSQC ranges from 10 to 40 min, depending on the sample concentration and the digital resolution needed. In addition, such experiments can be used only for proteins with very slow relaxation times, in the range of one to several hours. For instance, this method was successfully applied to wild-type ∆+PHS SNase and a series of variants having extremely slow relaxation time (up to 12 h) [65]. This drawback has been at least partially circumvented by methodological developments during the last decade: advances have been realized in the field of “real-times” measurement of NMR multidimensional experiments [66,67,68,69], extending the application of real-time 2D NMR. Now, 2D correlation experiments can be acquired in tens of seconds, and sometimes even in less than one second, instead of tens of minutes. For example, 2D [^1^H-^15^N]- SOFAST-HMQC experiments [67,69] recorded in two minutes have been used to monitor the kinetics of unfolding of Titin I27 domain (Figure 6), exhibiting relaxation times of about 30 min (see Figure 3B). Similar experiments, but recorded in only 25 s, have been used for the L125A variant of ∆+PHS SNase, with relaxation times shorter than 10 min [65]. The use of “ultra-fast” 2D NMR spectroscopy [66], allowing to record a 2D spectrum in only one scan, can in principle extend the use of real-time 2D spectroscopy to proteins with shorter relaxation times, in the minute range. In addition, fast or ultra-fast experiments can be used in combination with Non Uniform Sampling (NUS) methods [70,71,72], which can speed up data collection. These methods allow for a decrease in the total number of points (the number of FIDs) used for sampling the indirect dimension (^15^N dimension in the [^1^H,^15^N] HSQC experiments), maintaining the digital resolution at the expense of possible artifacts in the processed spectrum. Currently, a 4-fold gain in measuring time can be obtained, compared with the conventional method.

Obviously, the main limitation of this method remains the sensitivity, combined to a correct spectral resolution, of these experiments. In addition, playing with (increasing) the *P*-Jump amplitude in order to increase the sensitivity of the measurement, due to the subsequent increase in the intensity change for the cross-peaks, reaches also some limits. Indeed, the *P*-jump amplitude should remain moderate to avoid any imbalance between the folding and the unfolding reactions. Thus, an excessive positive *P*-jump will favor the unfolding reaction at the expense of the folding reaction, yielding erroneous values for the kinetic parameters. For instance, in the case of Titin I27, we have used pressure jumps of 200 bar, corresponding to about 10 percent of the pressure range needed to fully unfold the protein (2000 bar) [44]. 

Real-time 2D NMR spectroscopy remains inappropriate to study sub-second folding kinetics, which is the case for a lot of globular proteins. In the case of proteins with fast relaxation times (<1s), other NMR approaches are available, mainly based on 2D exchange spectroscopy techniques. The use of high-pressure ZZ-exchange experiments was introduced by Zhang et al. to obtain residue-specific folding rates for the two autonomous N-terminal and C-terminal domains of the ribosomal protein L9 [73]. This method is applicable to any proteins under experimental conditions where the folded/unfolded species exchange in a few tens to a few hundreds of milliseconds. 

More recently, Charlier et al. significantly improved the pressure jump apparatus originally designed by Kremer et al. for introducing pulsed pressure perturbation in 1D and 2D NMR experiments [51], allowing for the switching of pressure on a millisecond time scale [74]. Combined with adequate 2D heteronuclear NMR experiments, this system allows measuring the rate of exchange and chemical shifts of the folded, intermediate, and unfolded states.

## 5. Conclusions

While pressure perturbation allows one to finely and reversibly tune the stability of a protein and to modulate the rate of a conformational exchange, NMR spectroscopy can bring the spatial and temporal resolution necessary for the description of the protein folding energy landscape. Thus, HP-NMR, combining pressure perturbation, and NMR spectroscopy can give, at a residue-level resolution, an accurate structural and dynamical description of the protein folding energy landscape, revealing the existence of intermediate states as well as the rates of the associated local rearrangements.

Beyond this fundamental interest, a better understanding of protein folding/unfolding mechanisms is mandatory in many fields. Protein misfolding is involved in most of the neurodegenerative diseases, such as Alzheimer, Parkinson, prion disease, etc. The comprehensive study of the folding mechanism of the proteins specifically involved in these diseases [75,76,77,78] might allow the rational design of more efficient drugs [79]. Giving clues on the phenomena underlying protein stability, such studies can also be meaningful for the design of industrial enzymes able to work at high pressure. Thus, understanding how a protein can accommodate mutations to gain stability, keeping its function intact, has an important economic impact. Combining NMR with high pressure is an extremely powerful approach in this particular field, providing rigorous answers to important questions.

## Abbreviations and Nomenclature

Ig-like: Immunoglobulin-like, HP-NMR: High (Hydrostatic) Pressure Nuclear Magnetic Resonance, TSE: Transition State Ensemble.
